# *Galleria mellonella*: A model of infection to discern novel mechanisms of pathogenesis of non-toxigenic *Vibrio parahaemolyticus* strains

**DOI:** 10.1080/21505594.2017.1388487

**Published:** 2017-11-30

**Authors:** Diliana Pérez-Reytor, Katherine García

**Affiliations:** Unidad de Microbiología, Instituto de Ciencias Biomédicas, Facultad de Ciencias de la Salud, Universidad Autónoma de Chile. Llano Subercaseaux 2801, San Miguel, Región Metropolitana, Chile

**Keywords:** *Vibrio parahaemolyticus*, non-toxigenic strains, virulence, *Galleria mellonella*

## Abstract

*Vibrio parahaemolyticus* is a leading cause of raw seafood-associated bacterial gastroenteritis in the world. Its pathogenesis is likely to be multifactorial, although the most characteristic virulence-associated factors are the toxins TDH and TRH, in addition to the Type-III Secretion System-2, which codes for diverse effectors involved in cytotoxicity and enterotoxicity. However, diarrhea cases produced by clinical strains lacking all of these main virulence factors (non-toxigenic strains) have been reported in many countries and they can represent up to 9-10% of the clinical isolations. So far, although there have been significant advances in the description of the virulence factors of *V. parahaemolyticus*, the ability of non-toxigenic strains to cause illness is still not completely understood. To elucidate this question it is necessary to have adequate infection models. The susceptibility of *G. mellonella* to the infection with non-toxigenic strains seems to be the response to identifying new virulence factors and consequently providing new insights into mechanisms of the virulence of non-toxigenic strains. This new model means an invaluable contribution to public health, since the understanding of virulence in strains lacking the traditional major toxins is essential to detect these strains present in waters and marine products and avoid possible food-borne infection.

*Vibrio parahaemolyticus* is a Gram-negative, halophilic bacterium which naturally inhabits estuaries and marine environments, where it is able to persist and proliferate.^1^ This microorganism was reported for the first time as the etiological agent responsible for a gastroenteritis outbreak in Osaka, Japan^2^ during the fall of 1950. Today, *V. parahaemolyticu*s is a leading cause of raw seafood-associated bacterial gastroenteritis in the world^3^ although most of the environmental strains are non-virulent and only few strains have the ability to cause infection in humans.^4^ Estuaries and marine environments represent a broad reservoir of virulence-associated genes of the genus *Vibrio*.^5^ These genes may be combined by horizontal gene transfer in high frequency, and produce pathogenic species if they are incorporated in an appropriate background.^6^ As a consequence, bacterial pathogens continue to cause problems because of the emergence of new pathogens and the evolution of existing others. This is especially important because the diarrhea produced by *V. parahaemolyticus* is transmitted by mollusks and other seafood contaminated with environmental bacteria, thus human pathogenesis evolved by coincidental selection of traits beneficial for bacteria in the ocean that also conferred virulence in humans.^7^ Notably, bacterial evolution has led to the emergence of pandemic or pathogenic clones with expanded ecological persistence and dispersion, as occurred in 1996 with the emergence of *V. parahaemolyticus* pandemic strain serotype O3:K6, which caused thousands of clinical cases worldwide.^5^

The pathogenesis of *V. parahaemolyticus* is likely to be multifactorial,^8^ although the most characteristic virulence-associated factors are two toxins, the thermostable direct hemolysin (TDH) and the *tdh*-related hemolysin (TRH),^9^ in addition to the Type-III Secretion System present in chromosome II (T3SS2) which codes for diverse effectors involved in cytotoxicity and enterotoxicity.^10^ However, Jones and other researchers reported clinical isolates negative for the two hemolysins and T3SS2, indicating that the *tdh, trh* and T3SS2 genes are not necessarily predictive of pathogenic potential.^11,12^ Diarrhea cases produced by clinical strains lacking *tdh, trh* and T3SS2, called non-toxigenic strains because they lack the main toxins, have been reported in many countries;^13,14,15^ they can represent up to 9–10% of the clinical isolations.^15^ So far, although there have been significant advances in the description of the virulence factors of *V. parahaemolyticus*, the ability of non-toxigenic strains to cause illness is still not completely understood. Several theories have been proposed to explain why these strains are isolated from sick patients, including coinfection with pathogenic strains (ingested mussels contain a mixed population of bacteria including toxigenic and non-toxigenic strains), loss of virulence genes during or before the infection and the presence of virulence factors not yet discovered.^16^

To elucidate if the non-toxigenic strains have novel virulence factors involved in mechanisms of infection unidentified, it is necessary to have adequate infection models. However, the limited understanding of the pathogenesis of the diarrhea induced by *V. parahaemolyticus* to date could be explained in part because there are few analyses of human intestinal samples obtained from infected patients and because of the absence of animal models that mimic the human disease.^17^ Nonetheless, some models like orogastric and peritoneal mice, orogastric infection of infant rabbits and rabbit ileal-loop models have been previously used and the results obtained have provided information about intestinal colonization and pathogenesis.^18^ Zebrafish have been also used as an infection model for *V. parahaemolyticus*, providing a method to screen strains with varying virulence potential.^8^ However, the selection of experimental animal models for research is subject to bioethical aspects and the 3R (Replacement, Reduction and Refinement) criteria of Russell and Burch^19^ must be considered for this purpose. The use of a non-mammalian model to study *V. parahaemolyticus* allows high throughput screening of a large number of strains at low cost and reduces the dependence on animal models.

*Caenorhabditis elegans*, a free-living transparent nematode, has been used as a model of *V. parahaemolyticus* infection. This microorganism is able to persist in the intestine of *C. elegans*, leading to distention of the intestinal lumen. However, although the bacterium induces a strong inflammatory response in the host intestine, the detailed mechanism of pathogenesis is still unclear.^20^ Fortunately, Wagley and coworkers recently reported for the first time that *Galleria mellonella* is susceptible to infection with *V. parahaemolyticus. G. mellonella*, the larva of the great wax moth, has been investigated as an infection model, showing susceptibility to a wide range of fungi and bacteria^21^ including human pathogens^22^ such as *Pseudomonas aeruginosa, Enterococcus faecalis, Staphylococcus aureus, Yersinia pseudotuberculosis* and *Campylobacter jejuni.*

Interestingly, *G. mellonella* seems to be susceptible not only to a lethal infection with toxigenic clinical strains, but also to non-toxigenic clinical strains. Also, the moth does not develop the disease when treated with environmental isolates,^22^ suggesting that this is an excellent model to distinguish pathogenic from non-pathogenic strains. Some important differences can be observed in the pathogenesis of disease induced by toxigenic and non-toxigenic strains, suggesting that TDH and/or TRH are involved in detrimental effects on the infected larvae.^22^ So additionally this model may also be adequate to differentiate between toxigenic and non-toxigenic strains.

The susceptibility of *G. mellonella* to the infection with non-toxigenic strains would allow screening different genes through the use of bacterial mutants, with the purpose of identifying new virulence factors and consequently providing new insights into mechanisms of the virulence of non-toxigenic strains. As a matter of fact, this model in combination with whole genome sequencing data of diverse *V. parahaemolyticus* strains allowed Wagley and coworkers to observe that *mutT* (nudix hydrolase) mutant strains were unable to kill *G. mellonella*, suggesting a role of this gene in virulence processes.^22^ In light of the results of Wagley *et al.,*^22^ the model of *G. mellonella* to study the infection of *V. parahaemolyticus* would mean an invaluable contribution to public health, since the understanding of virulence in strains lacking the traditional major toxins is essential to detect these strains present in waters and marine products and avoid possible food-borne infection.
